# DARLENE – Improving situational awareness of European law enforcement agents through a combination of augmented reality and artificial intelligence solutions

**DOI:** 10.12688/openreseurope.13715.2

**Published:** 2022-01-21

**Authors:** Konstantinos C. Apostolakis, Nikolaos Dimitriou, George Margetis, Stavroula Ntoa, Dimitrios Tzovaras, Constantine Stephanidis

**Affiliations:** 1 Institute of Computer Science, Foundation for Research and Technology Hellas, Heraklion, Crete, GR-70013, Greece; 2 Information Technologies Institute, Centre for Research and Technology Hellas, Thessaloniki, GR-57001, Greece; 3 Computer Science Department, University of Crete, Heraklion, GR-70013, Greece

**Keywords:** Fight against criminality, Control and surveillance of areas, Augmented reality glasses, 5G network, Situational awareness, Deep learning, Computer vision

## Abstract

**Background:** Augmented reality (AR) and artificial intelligence (AI) are highly disruptive technologies that have revolutionised practices in a wide range of domains, including the security sector. Several law enforcement agencies (LEAs) employ AI in their daily operations for forensics and surveillance. AR is also gaining traction in security, particularly with the advent of affordable wearable devices. Equipping police officers with the tools to facilitate an elevated situational awareness (SA) in patrolling and tactical scenarios is expected to improve LEAs’ safety and capacity to deliver crucial blows against terrorist and/or criminal threats.

**Methods:** In this paper we present DARLENE, an ecosystem incorporating novel AI techniques for activity recognition and pose estimation tasks, combined with a wearable AR framework for visualization of the inferenced results via dynamic content adaptation according to the wearer’s stress level and operational context. The concept has been validated with end-users through co-creation workshops, while the decision-making mechanism for enhancing LEAs’ SA has been assessed with experts. Regarding computer vision components, preliminary tests of the instance segmentation method for humans’ and objects’ detection have been conducted on a subset of videos from the RWF-2000 dataset for violence detection, which have also been used to test a human pose estimation method that has so far exhibited impressive results, constituting the basis of further developments in DARLENE.

**Results: **Evaluation results highlight that target users are positive towards the adoption of the proposed solution in field operations, and that the SA decision-making mechanism produces highly acceptable outcomes. Evaluation of the computer vision components yielded promising results and identified opportunities for improvement.

**Conclusions:** This work provides the context of the DARLENE ecosystem and presents the DARLENE architecture, analyses its individual technologies, and demonstrates preliminary results, which are positive both in terms of technological achievements and user acceptance of the proposed solution.

## Introduction

In recent years, increasingly higher levels of technological advancement have brought upon rapid change in many facets of modern society, including the digital transformation of law enforcement agencies (LEAs) around the globe. Technologies, such as augmented reality (AR) and machine learning (ML) are being leveraged in order to endow humans with superior, computer-like capabilities, elevated situational awareness (SA) and an enhanced capacity to accomplish a vast variety of tasks and assignments. Both AR and artificial intelligence (AI) are rapidly evolving technologies that often go hand in hand and have greatly matured in recent years, moving from laboratory prototypes to mainstream applications. AR aims at the presentation of virtual components or other computer-generated content along with real life scenes. To this end, AR devices are used to render virtual content overlaid on the user’s field of view or on captured imagery. Indicatively, such devices can be handheld, like smart phones or tablets; head mounted displays (HMDs) in the form of glasses or visors; or projectors. In this way, AR complements the human capacity to gather information about the real world with superimposed 2D and 3D objects and graphics. As such, AR can be a potentially transformative technology for security and policing functions, one that can deliver disruptive innovations to future smart policing (Policing 4.0 ^1^). AR alone however cannot deal with the complex task of scene interpretation, as it is a technology primarily focused on how to optimally combine digital and virtual information in one seamless representation. Therefore, AR applications are often tightly coupled with AI and particularly with computer vision (CV) technologies, toward the registration of 3D models, instance segmentation and 3D pose estimation. For instance, an object has to at least be segmented in the user’s field of view, or in more complex scenarios, a precomputed 3D model has to be registered on the captured video stream in order for the application to properly superimpose information about the object and display it in a seamless manner to the user through a heads-up display (HUD) application. ML becomes a useful tool in such cases, lending itself towards analysing large volumes of data acquired by multiple sensors, and can be optimised for real time inference, enabling assessments to be provided faster and in a much more coordinated manner in comparison to existing conventional approaches, which rely only on tactical protocols and human capabilities. With the advent of deep learning (DL), AI has achieved impressive results that often surpass human performance in typical tasks, having found several commercial applications for data analysis indicatively, in medicine, media production and surveillance. In the domain of security, AI’s ability to sort through massive volumes of data has enabled the simultaneous analysis and accurate inference of multiple intensive data stream (e.g. video feeds).

Together, AR and AI technologies can aid police officers who are forced to make difficult decisions in high-pressure situations, delivering crucial information in a way that is instantly applicable to the situation in progress. This is expected to significantly boost officers in outmanoeuvering any potential adversary. Advantages like this can be achieved by first analysing real world scenes and real-time data through DL-based techniques and components, and subsequently utilising virtual graphical elements (such as textual annotations) in a coordinated real-time presentation tailored for police use through wearable, binocular AR smart glasses (ARSGs). Nonetheless, both AR and AI have still several open challenges that need to be addressed. This is why in the context of smart policing, AR and AI have only found limited application in security operations centres (SOCs), tasked with communicating analysis results to officers operating in the field. Trying to pinpoint the barriers for broader adoption, an obstacle lies on the computational requirements of AI analysis, which requires substantial infrastructure for data processing and storage. In addition, sensible AR applications meant to be worn by patrolling officers present strict requirements for real-time rendering and user interaction, all while operating on a limited computation budget to prevent device overheating. Subsequent, this process increases delay contradicting LEA officer requirements for real time analysis, adding further requirements for an adequate communication infrastructure for fast and efficient data transmission. Additional obstacles are further introduced when the human factor (i.e. analysing AI data from multiple data stream can become overbearing for human operators, leading to critical pieces of information being neglected during team communication) and ethical concerns are implicated, as ARSGs are wearable computers equipped with a mobile internet connection ^1^ with the capacity to record video and audio of people in stark contrast with privacy laws recently put in place by the EC.

In this paper, we present DARLENE, an EU-funded Research and Innovation Action, which aims to accelerate the development and take-up of purpose-built ARSGs for LEAs, with the aim being to deliver elevated SA for police officers responding to criminal and terrorist acts. The solutions targeted will be designed to assist in more efficient decision-making, especially during such high-stress situations, where police personnel are forced to make quick decisions and remain one step ahead of adversaries of all sorts. The goal is to develop the software and hardware necessary to enable the use of ARSGs for the presentation of AI analysis results and the real-time processing along the entire computation continuum comprised of edge, fog and cloud resources. LEAs will hence be able to rapidly identify illegal goods and activities, being shown a hyper-virtual representation of their surrounding environment, aimed at alarming them to imminent dangers during response to high-risk situations, such as e.g. active shooting incidents. DARLENE will hence enable LEAs to reduce and prevent crime, as well as more quickly respond to crimes already in progress, by enabling them to sort through massive volumes of data to predict, anticipate and prevent criminal activities, make better informed tactical decisions and provide enhanced protection services for European citizens.

DARLENE incorporates the advantages offered by both AR and AI to realize technology-assisted policing in ways that have previously only been imagined in science fiction. Police officers will patrol and respond to incidents using wearable gear that will utilize AI in the form of ML and DL routines to enable rapid scene analysis and interpretation to capture, outline and single-out interesting findings and threats requiring the attention of the smart glasses wearer. AR will then be used to superimpose such mission-critical information directly on top of the real world, catering to the officer’s unique point of view, and ensuring a functional visualization experience, meant to enhance the officer’s capacity to respond to incidents. To validate DARLENE’s vision, the technology will be put to the test in real-life LEA operational scenarios, where the goal is to demonstrate the superior performance expected by officers donning our solution in contrast to unequipped individuals responding to a similar threat scenario.

To allow European LEA practitioners to acclimate to the proposed solution, extensive training and demonstration activities are planned throughout the lifetime of DARLENE. These are aimed at empowering police officers with the training, equipment and tactical capabilities required to respond to terrorist, or other kinds of physical attacks, along with contributing to the development of LEAs’ threat assessment skills. Further, the project will work on security and ethics by design approaches, taking care so that infrastructure setup and privacy preserving technologies are tightly integrated. In this regard, DARLENE will utilise edge processing and real-time algorithms in order to limit data storage and transmission, thus avoiding relevant privacy issues and cyber-threats. Following this approach, DARLENE aims to contribute in improving the confidence of citizens towards lawful use of AR technologies in law enforcement and public safety situations, ensuring that the appropriate consideration and attention towards an ethically sound approach to AR smart glasses for law enforcement is properly applied. This will be further constituted by a carefully planned, effective involvement of the relevant actors (end users) and stakeholders across all project activities undertaken throughout the lifetime of the project, from requirements elicitation to experimentation and validation.

The remainder of this paper is organised as follows: initially, an overview of the current situation for AI and AR technologies and their use in law enforcement is provided. Then, the overall DARLENE concept is presented, before a detailed technical specification of the system, along with envisioned use cases are presented in the next section. Following, the planned breakthroughs regarding AR, AI and communication infrastructure which substantiate the DARLENE long-term vision are presented. Then, the methods for the preliminary assessment of the overall DARELENE concept and its technological components are introduced, followed by the presentation of results. Finally, a discussion on results and a look onto future work in the context of the project’s lifetime is presented, before conclusions are drawn in the last paper section.

## AI & AR technological landscape for the security and safety sectors (related work)

### Smart wearables and AR for the security and safety sector

The constant evolution of modern security threats and linked challenges have forced law enforcement agencies (LEAs) in Europe to assume increasingly diverse roles, which require officers to develop similarly diverse sets of skills. As an additional burden, the capacity of EU member state police forces to counter both terrorist organisations as well as unknown, lone individuals and criminals or criminal networks has suffered a severe blow due to the recent sovereign debt crisis forcing expenditure cuts on police budgets, which in some cases have been ongoing for several years. With that in mind, affordable augmented reality (AR) technology can dramatically impact police work, opening up new and innovative means by which to combat criminal and terrorist acts. Toward this end, ‘Wearable Augmented Reality Devices’, or ‘Augmented Reality Smart Glasses’ (ARSGs) ^2^ could deliver significant impact on the effectiveness of police process.

Already in the early 2000s, AR was heralded as having the potential to be a transformative technology for policing. In a 2003 FBI report, Cowper & Buerger examined implications of AR for use as a law enforcement tool, estimating that a single officer equipped with AR technology would be able to carry out the same amount of work as three unequipped individuals ^3^. Indeed, use cases for police functions include both obvious upgrades to the profession (e.g. matching individuals in crowds against a database of known suspects ^4^; scanning licence plates on vehicles ^5^; training for crime scene investigation ^6^ as well as less-obvious applications (e.g. employing AR for real-time translation ^7^ with the aim to increase individual functioning of police officers and facilitate a more efficient response to crime. Twelve years later, in a 2015 report, ABI Research estimated that police, along with military, security, warehouse, and bar code scanning operations would amount to approximately 90% of smart glasses sales ^2^. Reports of smart glasses being, or having been employed for law enforcement in several countries both outside (e.g. China ^8^, Abu Dhabi ^9^) as well as within the EU (Netherlands ^10^) have surfaced, while a healthy amount of work on wearable AR in service to law enforcement has been carried out in recent years. For instance, the TARGET project employed the use of AR wearable visualization tools toward the effective training of European security units in counter-terrorism and mission-critical events ^11^. In a similar manner, the AUGGMED project developed a serious game platform to enable single- and team-based training of LEAs using Virtual and AR enablers ^12^. Most recently, ARESIBO aims to employ the use of wearable AR to improve border and coast guard surveillance functions. Despite these endeavours, and the encouraging findings that have surfaced as a result however, police adoption of ARSGs within the EU remains strikingly low to this day.

The most prominent reason for this stems from ASRGs yielding data protection implications similar to that of closed-circuit television (CCTV) and dash-cams. After the adoption of the General Data Protection Regulation (GDPR) by the European Parliament and the Council of Europe in May 2018, strict principles for fair and lawful processing of personal data have been laid down, including transparency, valid legal base for processing operations, purpose limitation, data minimisation, limitation of data retention, data quality and security, rights of the data subjects and independent supervision. As such, while ARSGs may be useful tools in a variety of application areas, their use can be controversial in that they yield a high potential to undermine the privacy of individuals. Especially concerning the use of ARSGs by LEAs, and the integration of sensing hardware, such as cameras and microphones, AR gear is seen as having the capacity to record video and audio in a discreet manner, raising the issue that people being recorded may actually not even be aware of it. The image of a person constitutes personal data within the meaning of Article 2(a) of Directive 95/46/EC ^3^ and of Article 4(1) of the GGDPR ^4^, inasmuch as it allows for the identification of individuals, and in the same way as image and sound data recorded by means of CCTV (closed-circuit television) and other video surveillance systems are considered personal data. Furthermore, smart glasses may suffer from security loopholes and be prone to hacking, as is the case with all internet connected, Internet of Things (IoT)/wearable devices, thus exposing exploits for the theft of data or the execution of unauthorised software ^13^. Hence, the use of ARSGs for law enforcement activities within Europe should lend appropriate consideration to privacy laws, as well as the data protection standards adopted by the European Union.

DARLENE aims to mitigate these concerns through the real-time operation of the ARSG/ artificial intelligence pipeline that allows ignoring data irrelevant to a security threat, without the need to first store such data for offline processing. In this regard, the real-time performance of DARLENE algorithms, aside its obvious benefit for rapid decision making, will also limit data recording and storage requirements for offline processing, thus alleviating ethical concerns related to storage and handling of massive data volumes. From the same viewpoint, processing data on the edge will not only ease up communication requirements and decrease latency, but will enable *in situ* data processing thus avoiding privacy threats from cyber-security attacks (e.g. eavesdropping) during data transmission and cloud processing. Additional provisions will be made throughout the project’s lifetime by identifying relevant overarching legal rules and ethical principles and ensuring compliance of the final technological solution developed with the EC regulatory framework as well as the recommendations of policymakers and stakeholders in the security domain.

### Artificial intelligence for the security and safety sector

Artificial intelligence (AI), and particularly machine learning (ML) and its emerging subset of deep learning (DL) algorithms play an important role in security functions due to its proactive threat mitigation capabilities. Particularly, computer vision methods based on DL have shown impressive results reaching human-level performance in several tasks. One such task is instance segmentation, which combines detection of an object with identification of its contour in the scene. Such methods have immediate application in surveillance as they can be used to isolate humans from the background or pinpoint objects such as weapons and unattended bags. In this line of research ^14^, introduced the moving-object proposals generation and prediction framework (MPGP) to reduce the search space and computation time while extracting better objects proposal. Another interesting method, focusing on pedestrian detection has been presented in 15 here the proposed convolutional pedestrian detection model can detect humans far away from the camera centre utilising a novel auto-zooming mechanism. S4Net by 16 is another method that achieves real time performance with low resolution images by jointly using local and surrounding context enabling instance segmentation even with occlusion. A related task that often requires instance segmentation of humans as a prerequisite, is pose estimation which aims at localizing parts of the human body to derive a skeletal pose representation. For this task, a promising method ^17^ has been introduced using a deep convolutional architecture that maintains the original image resolution in parallel with low resolution network branches while a follow-up work ^18^ has improved pose estimate results for persons in low scale.

These categories of methods, namely for instance segmentation and pose estimation, have great interest for augmented reality (AR) applications as they can be used to analyse the user’s field of view and properly add visual content. They are of high importance for the security sector as well as they can be used to identify friend or foe (IFF) and understand abnormal activity ^19^. For human action recognition in surveillance videos, one of the most influential DL approaches ^20^, uses a 3D convolutional model to capture both spatial information and motion in consecutive frames. In another interesting work ^21^, the proposed method aims for detecting abnormal crowd activity related to panic and escape behaviour related to violent events and natural disasters, introducing motion information image (MII) to encode optical flow and train a deep model to detect abnormal events. In this line of work ^22^, introduced an AI framework using edge processing for the timely detection of petty crimes using motion features for activity recognition. Forensics is another security domain where AI has found application with DL methods for face recognition having on-par performance with forensic examiners ^23^. Gait recognition methods have been also introduced that can be distantly applied from afar without the subject’s awareness. Indicatively ^24^, proposed the JRC-CNN gain recognition method that resolves viewpoint dependence and puts emphasis on motion of connected body joints while ^25^ combines a shallow convolutional network with a long short-term memory model to extract more efficient spatial and temporal features for gait-based human identification.

Aside their great academic merit, similar AI methods have been used in commercial products as in the case of Hikvision AI-enhanced cameras for facial recognition and vehicle identification as well as in larger integrated systems in the context of research projects. For instance, the European research project P-REACT developed a platform for the detection and archiving of petty crimes from video and audio streams. It implemented a hybrid approach that combined edge processing for light-weight analytics ^22^ and cloud computing for more complex processing ^26^. In another interesting European research initiative, the ALADDIN project has developed a multi-sensorial framework that uses AI techniques for the detection and localisation of unauthorised unmanned aerial vehicles (UAVs) while also exploring neutralisation techniques that have been integrated in a command and control system. Among other advances, traditional computer vision (CV) tasks for detection and segmentation have been enhanced with other sensors (e.g. radar) and deployed is use case of UAVs. Also in the security domain, the ASGARD research project followed an agile approach to close the gap between researchers and law enforcement agency (LEA) practitioners, offering tools, including AI methods, for multi-modal data processing focusing both on forensics and intelligence for crime prevention and anticipation. Another European project, PREVISION explores ML and visualisation methods for the analysis of heterogeneous big data streams in order to help LEA officers address physical and cyber security threats while the AIDA project is developing AI methods that will be integrated in a data analytics platform for the detection and analysis of cybercrime and terrorist threats. Finally, in INSPECTr, researchers are creating a shared intelligence platform incorporating AI and blockchain technologies to investigate cybercrimes and facilitate cross-border LEA collaboration.

Following this brief but representative state of the art analysis, it is obvious that AI solutions have started to find their position among technologies that LEAs use in their daily operations. However, it is also accurate to state that AI analysis is mainly applied on static data sources (e.g. wall-mounted CCTV cameras) while the complexity of the deployed methods often requires off-line processing on the cloud. DARLENE aims to close this gap, bringing AI capabilities to the officer in the field and seamlessly exploiting the entire computing continuum (i.e. edge, fog, cloud) with minimal latency via 5G technology. Another significant novelty of DARLENE lies on its cross-disciplinary approach, combining AI with AR, where visualisation of AI results is performed on augmented reality smart glasses in order to enable tactical LEA teams or officers in the field make informed decisions on each step of a police operation.

## DARLENE concept

Police officers in the field have to deal with emergency situations that can range from petty crime incidents to large scale crises. In such a context, rapid and correct decision making is vital to get the best possible outcome and ensure officers’ safety. A prerequisite for this is to have a complete picture of the ongoing incident or achieve situational awareness (SA) as is stated in police operation terms. Traditionally, officers relied on their instinct and experience but as technology matured, these factors have been reinforced with information from the respective security operations centre (SOC) that is delivered via audio wireless communication. The sources of information can be video feeds from surveillance cameras or information collected from other officer or civilians that are analysed in the operation centre and then communicated to the officers in the field ^27^. However, there are several limitations in this process that cause critical information to be overlooked or delivered too late. First of all, since information is aggregated and routed from a human operator in the SOC, there can be delays from the massive volume of data that has to be examined, which may also cause important pieces of information to be neglected. The means of communication from the operation centre to the field officer via audio communication can also fail to properly convey the intended information. Furthermore, aggregating information from multiple sources in short sentences that can be communicated adequately and comprehended fast by an officer in the field requires the combination of advanced communication skills and extended experience, which is quite challenging in the given operational context. Finally, current means of communication do not take into account individual officer characteristics, such as their age, gender, expertise (i.e. years of service in the force), characteristics regarding situational conditions and challenges, but also specific stress symptoms, individual preferences and needs, communication styles, emotional, physical and cognitive condition, etc. For instance, stress-related conditions can affect men and women in different ways, leading to fundamental differences in the way men/women adhere to the recommended solutions ^28^. These differences may be due to several factors, including biological determinants and psychological characteristics such as differences in coping styles. Any solution targeting the aforementioned challenges should therefore take into account these specificities when designing a real-time communications solution for law enforcement agents (LEAs) in the form of a graphics-based Heads-Up Display for boosting SA.

DARLENE is envisioned as a policing hardware and software ecosystem, one that consists of a wide range of technological components. Its aim is to deliver a significant boost to the police officer’s SA and support in tough decision-making, especially under loads of stress. The fundamental components of the DARLENE ecosystem will consist of a wearable micro-computer module, capable of delivering performance and power efficiency needed for demanding visual computing applications, and a head-mounted display unit embedded with tracking and sensing hardware, capable of running virtual 3D rendering applications in real time. The wearable system will accommodate secure, high performance networking with a Cloud-based infrastructure through a portable wireless, mobile access point. On the Cloud, DARLENE will host a computational setup for intensive machine learning (ML) pipelines, such as semantic and instance segmentation models, multi-person 2D and 3D pose estimation and anomaly detection mechanisms. Complementing the wearable end of the DARLENE overall concept, smart bands will be handed out to field operatives as a means to obtain and monitor physiological status. Physiological signals from the wearables will further feed into a personalisation service, whose aim will be to tailor the on-screen visuals to the most appropriate configuration with respect to the officer’s personal preferences as well as the situational context.

Exploiting technologies for positive security benefit is key in order for LEAs to stay ahead of adversaries of all sorts, and thus DARLENE aims to boost field agents’ SA, as well as enhance their ability to accomplish a number of dangerous assignments utilizing state-of-the art augmented reality smart glasses (ARSGs) and Cloud artificial intelligence (AI) technology to accomplish rapid scene analysis. To achieve this, DARLENE will analyse and interpret both real-time optical and sensor image input from the ARSGs worn by the police officers, as well as information gathered from intelligent surveillance environments, e.g. computing systems embedded within actual physical spaces, and networked through wireless communications links. Through this data, scalable, 3D maps of the operations area will be created, leveraging on automatic sensor readings that enable approximate calculations of both distance and height. These will be utilised in a two-fold manner. On the one hand, in the SOC, where enhanced cohesiveness between fellow ground units and better coordination with command and control personnel is a highly sought-after capacity. Secondly, the maps will be utilised by 3D rendering software running on the ARSGs, delivering a hyper-virtual representation of the environment to the wearer so as to aid in the location and apprehension of criminals, even when line of sight is broken due to obstructions. In this way, individual operatives are expected to benefit from digital overlays of virtual images onto their real-world field of vision, enhancing their capacity to respond to dynamic and potentially dangerous incidents. In such cases, real world objects and people will be highlighted in real time, thus effectively speeding up the process of identification (whether this refers to detecting firearms or armed perpetrators). DARLENE thus represents a combination of cutting-edge ICT technologies, taking advantage of the fusion of massive heterogeneous data originating from ARSGs, wearable sensors and infrastructure Internet of Things (e.g. surveillance cameras) towards delivering personalised context-aware recommendations and predictions, which anticipate and prevent criminal activities, (self)injury and fatalities.

**Figure 1.**
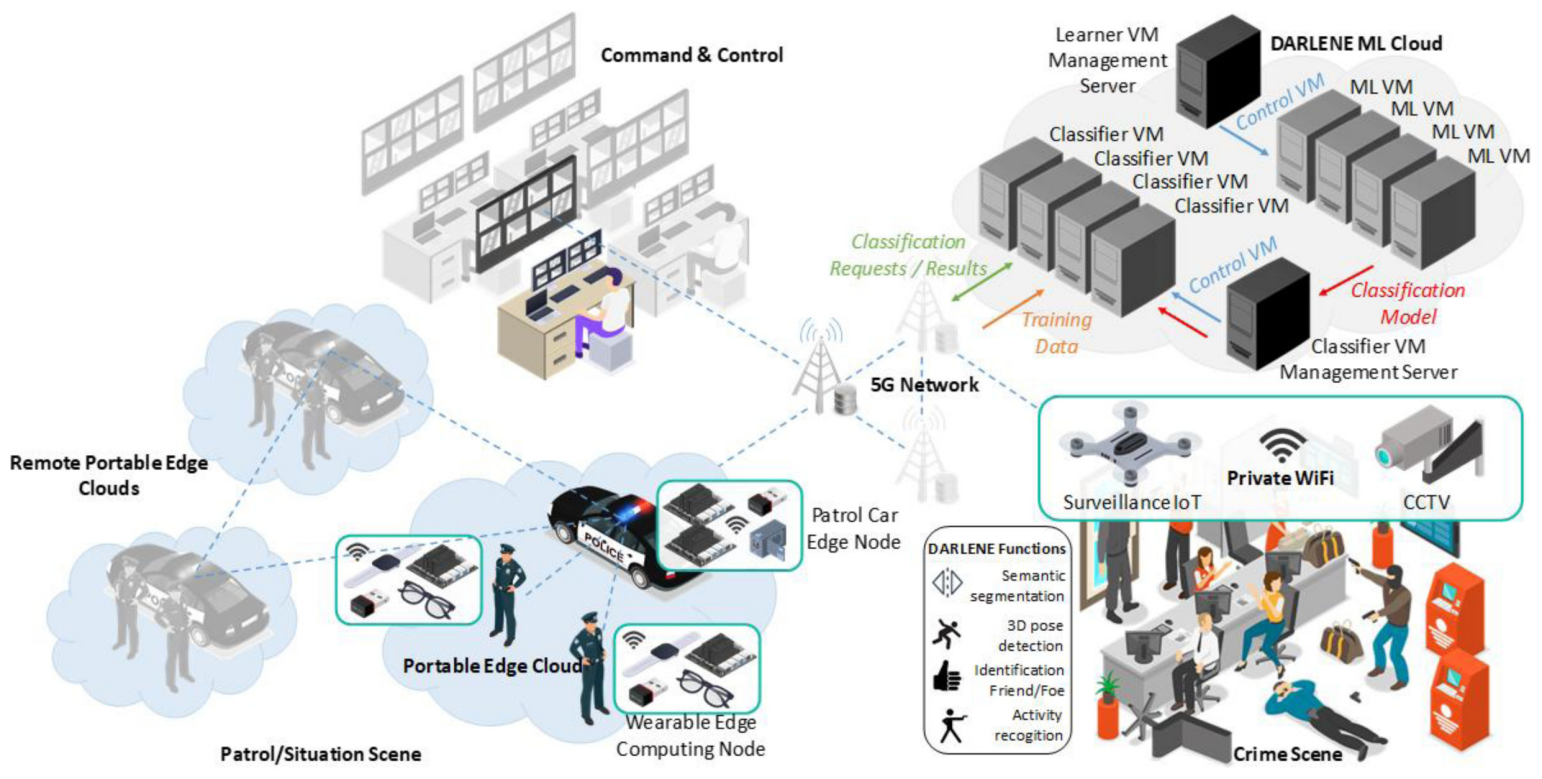
DARLENE overall concept. CCTV=closed-circuit television, ML=machine learning, VM=vision modules, 3D=3 dimensional.

The DARLENE concept described above and graphically illustrated in Figure 1, is based on high-performance connectivity as a key enabler, where ARSGs, portable micro-computers and even additional computing devices (for instance, installed on patrol cars parked at the scene) generate a private local area network for edge computing. This networking infrastructure will be wholly optimised in preparation of the coming 5G era. It will aim at distributing the computational load of ML and computer vision applications optimally, with the ultimate goal being to shorten the distance from data centres to the end users, by having several base stations working simultaneously to provide efficient computation and connectivity transfers to the ARSGs. This distribution will be performed by DARLENE software specifically designed to orchestrate and manage network resources in an automated manner, toward delivering a practical federated learning (FL) paradigm. In this respect, each “edge area network” will be responsible for running optimisation algorithms based on the acquisition of new local data. New learnings obtained from the algorithms running on the AI (micro-)processing modules will then be sent to the DARLENE ML Cloud on an encrypted channel, where updates will be aggregated with centralised algorithms, allowing new data from each scene to be integrated and algorithms to treat them as if being directly trained on these data. As such, the ML Cloud will re-distribute an updated model to each edge area network, accumulating all learnings made from all LEA users, whilst remaining personalised to each individual case.

## DARLENE ecosystem

### Requirements extraction

From the early stages of the project an elaborate system specifications analysis process has been put forward, in which the DARLENE system and end users’ requirements have been elicited and analysed. The outcome of this process provided us with the functional and non-functional requirements that constitute the implementation guide for the technical components of the DARLENE platform, as well as a main pillar for the DARLENE architecture specification elaborated in the next sub section. In specific, several co-creation sessions were carried out with the participation of members of law enforcement agencies (LEAs) partaking in the DARLENE project acting in a supporting role. Specifically, 30 individuals working at LEAs across Europe contributed to these workshops with their ideas, desirable features and requirements pertinent to their organizational needs. The workshops were held toward better understanding those desired functionalities and capabilities, matching them to capabilities that the DARLENE smart glasses solution should provide, and eventually leading up to the design of a first set of DARLENE user interface component prototypes based on those requirements. In a secondary activity, an analogy was defined between the elicited designs and requirements to the machine learning algorithms and components in the platform architecture, so as to make sure that the architecture functional blocks are properly matched against the system desired functionalities.

### Architectural overview

**Figure 2.**
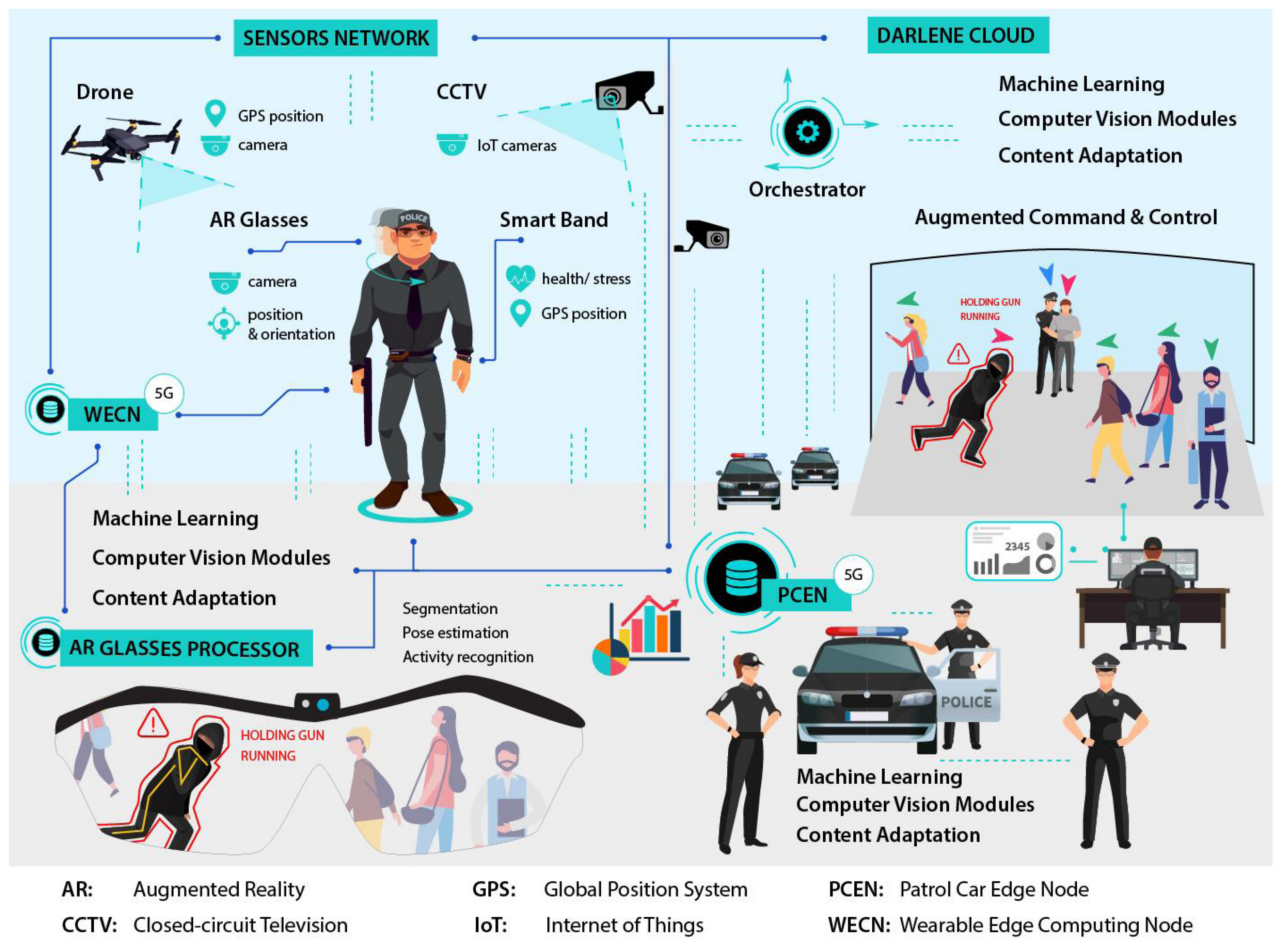
DARLENE architectural overview.

DARLENE follows a modular architecture distributed along the computing continuum. It contains an expandable Internet of Things (IoT) network of sensors and a hierarchy of computational layers on the edge, fog and cloud that can perform progressively more complex functionalities, mainly by artificial intelligence (AI) and visualisation modules. Communication between layers is performed via high-performant cellular networking (5G) in order to handle intensive, live data streams from the IoT network that require high bandwidth and minimal latency. In this respect, a research direction that DARLENE explores is the dynamic allocation of 5G network resources according to the urgency of the situation that police officers face, thus exploiting the results from AI algorithms for activity recognition and stress level analysis in order to guide resources allocation. For example, DARLENE will allocate more resources for an officer dealing with an ongoing crime or facing a severe security incident (e.g. shooting) prioritising if needed over officers on regular patrol duty. Data streams are collected from the Sensors Network, constituting the first layer of DARLENE architecture as depicted in Figure 2, which includes wearable and static IoT devices. Wearable sensors on a law enforcement agency (LEA) officer include the camera and the global positioning system (GPS) sensors on the augmented reality (AR) smart glasses (ARGS) as well as biometric and positioning data from a smart band. Additional video streams are coming from unmanned aerial vehicles cameras or static CCTV cameras that can optionally be registered on a 3D model of the building where an operation is taking place.

Moving to the computational layers of DARLENE, the AR glasses processor (ARGP) and the wearable edge computing node (WECN) reside on the edge side. The ARGP is a processing layer responsible for the adaptive visualisation of various types of information on the visor of the ARSGs. It is directly connected to the WECN, which is a small autonomous computational unit that the officer carries along with the rest of his/her gear. WECN has several machine learning (ML) and computer vision (CV) capabilities for the *in situ* analysis of data streams primarily from the camera on the ARSGs and data from a smart band that the officer wears; and secondarily, from nearby cameras. The WECN has a CV component for the semantic and instance segmentation in incoming video streams component that will separate the different objects in a scene from the background, and identify their class. Also it includes a pose estimation module for the detection of body joints and the estimation of the skeletal configuration for each person in the field of view of the ARSG camera. Pose estimation is supported both in 2D and 3D where the pose of humans will be inferred with respect to a global coordinate system (GCS). Activity recognition will analyse the actions of humans in order to understand their activities. The content personalisation module consists of the adaptation manager, stress level analysis and LEA profile database. The adaptation manager decides on the level of information that will be projected on the ARGS according to an officer’s stress level and the context of a situation as it will be recognised by the activity recognition component. To this end the adaptation manager will select the most appropriate visualisation template that will be stored in the LEA profile database. Since deep learning methods usually require a huge amount of training data that can be time-consuming to annotate, DARLENE has dedicated ML modules that deal with data scarcity and can support new operational requirements. In particular, active and few-shot learning modules can support detection and segmentation of new object classes with few annotated examples. This way DARLENE is able to adapt faster to new user requirements compared to a traditional ML pipeline where a well-prepared dataset is needed. In the same direction, federated training is also supported, in order to off-load training from to the cloud to edge when possible. With federated training, DARLENE will be able to more easily adapt to new user requirements even without constant, high throughput connection to the cloud.

On the fog side of DARLENE lies the patrol car edge node (PCEN), essentially facilitating and coordinating multiple WECNs. The ML and CV functionalities in the PCEN are similar to the WCEN although more complex AI models will be supported as more computational resources are available. In addition, content personalization does not refer to individual users but to forwarding adaptation suggestions according to a more complete picture, which takes into account information from multiple WCENs. PCEN can also play the role of the central node for model transmission and update during federated training. Finally, the DARLENE cloud has more computationally complex and accurate ML and CV analytics and also introduces a new component for augmented command and control (C2). This component is responsible for the visualisation on all analysis results on a 3D interactive map of an operational site. Concretely, this map will be based on a 3D model of a site and will provide live information of the position, pose and status of LEA officers and other individuals, both friendly and hostile as identified by CV and ML components. For the creation of the 3D model, DARLENE cloud has a dedicated reconstruction module that can compute a 3D model of a site (e.g. multi-storey building) prior to an operation using 3D sensing, but can also import pre-existing 3D models. The C2 component has also the necessary user interfaces for visualizing more details on particular individuals on site and communicating with LEA officers.

### Foreseen use cases

DARLENE will combine cutting-edge technologies with ground-breaking research, exploiting augmented reality and machine learning in conjunction with other potentially disruptive technologies (e.g. emerging 5G networks) to deliver a means for law enforcement agents (LEAs) to exercise superiority in surveillance and tactical manoeuvering scenarios. To this end, a significant amount of work will be devoted to the co-developing of operational scenarios with LEAs participating in the project consortium, which will clearly illustrate the circumstances under which the DARLENE tools would operate. This process will further define the experimental environment in which the developed prototype will be tested, and subsequently evaluated on its merits to satisfy the elicited operational requirements during a later stage in the project’s lifetime. Two foreseen use case scenarios are being contemplated for the DARLENE ecosystem, including: i) rapid scene analysis for anomaly detection; and ii) tactical neutralisation of human adversaries in the presence of friendlies.

### Rapid visual scene analysis for anomaly detection

The current situation in surveillance relies on streams of images, taken from security cameras, which feed data to a control room video management software. For such use, augmented reality (AR) boasts a significant track record in surveillance applications as a means to guide the attention of the operator to a particular location on the screen ^29^. This however provides little to almost no information to the security personnel on the ground, who have to operate solely on verbal communication received through a security operations centre (SOC). Hence, performance of such operational teams relies on the efficient exchange of information between actors ^27^. In such cases, mission-critical information can be loosely tied, may be prone to misinterpretation and human error (e.g. coming from a distressed SOC operator), which in turn could risk opening points of vulnerability, or even jeopardising ground personnel safety.

**Figure 3.**
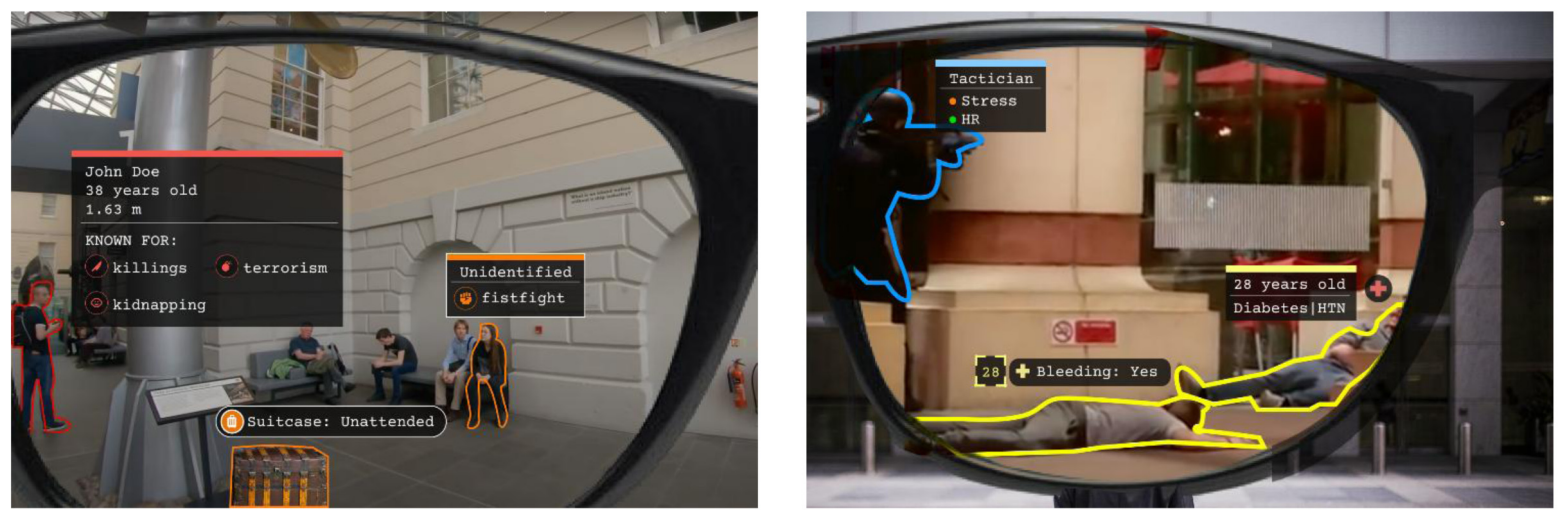
UC1 mock-up screenshots.

Airport police units carry out a variety of security functions pertinent to this task, with patrolling, investigation, control and response to emergencies being top among personnel responsibilities. Hence, DARLENE solutions will be employed to cover a wide range of security and control issues closely aligned to airport police directives, including: i) crisis management and healthcare provision after catastrophic incident (e.g. terrorist attack), where police officer duties include delivery of care and assistance to injured civilians; ii) criminal/terrorist apprehension, where, for instance, weapons and armed suspects are highlighted so as to immediately stand out, especially in crowded areas; and iii) rapid identification of abandoned/unattended baggage and potentially dangerous goods (e.g. explosives) during patrol operations. Through DARLENE, surveillance scenarios in the crowded areas of the airport can benefit from more accurate information exchange between teams of mobile security agents and airport SOC operators, thereby contributing to their collective situational awareness (SA). Throughout these scenarios, the AR smart glasses will highlight context-specific elements discovered in the scene after performing image analysis through the machine learning (ML) modules residing on the DARLENE ML Cloud, thus enabling security control teams to more quickly build knowledge of the situation. This in turn is expected to lead to better decision-making based on enhanced SA, allowing for airport police units to more efficiently address specific tasks pertinent to their mission. Measurable success criteria will include reduction to both the *mean time to detect* (the average time it takes the patrolling officers or SOC to become aware of a potential security incident) as well as the *mean time to resolve* (average time it takes the patrolling officers or SOC to remediate a threat after it has been discovered). To realise these scenarios, DARLENE will facilitate one of the projects pilots in the Larnaca International Airport (LCA/LCLK), as the Cyprus airport security directorate of Cyprus police is a collaborator on the project consortium. Figure 3 shows three mock-up screenshots illustrating envisioned functionalities for airport police patrol units.

### Tactical neutralisation of human adversaries in the presence of friendlies

Situational awareness (SA) is a critical skill to the survival and safety of police officers and community members alike. As such, it is mandatory for police officers to continuously work towards cultivating and improving a SA mind-set. Integrating advanced technology into such a way of thinking, police officers are expected to gain considerable advantages when dealing with dangerous situations, particularly ones that involve human adversaries. The basics of SA can be described in what is called the OODA loop, an acronym for the cycle ‘Observe, Orient, Decide, Act’ ^30^. This approach clearly favours agility over brute force in dealing with human adversaries, devising a response based on making better and faster decisions in order to outmanoeuvre the opponent.

With that in mind, this use case will address coordinated response and planning of tactical approach of law enforcement agents (LEAs) in active shooting scenarios, particularly in indoor crowded areas (e.g. transit stations, hotels, malls, banks, etc.). In such situations, police units are often at a disadvantage, as perpetrators have usually devoted a significant amount of time surveilling their target (‘Observe’); have familiarised themselves with their target’s security protocols, day-to-day activities and procedures and have devised counter measures (e.g. which weapons to use, which exits to cover, etc.) based on their observations (‘Orient’); have facilitated a plan of approach (‘Decide’); and are carrying out their plan step-by-step in pursuit of their goals (‘Act’). As such, adversaries are usually already in the fourth step of the OODA cycle when LEAs arrive at the scene. DARLENE will enable LEAs to take decisive and effective action that will enable them to catch up faster.

Utilising an array of imaging technologies from facilities’ Internet of Things resources (e.g. CCTV) or police-deployed equipment (e.g. surveillance drones) DARLENE will collect data in real time to structurally model 3D indoor maps with multiple floors and rooms, complete with semantic information for security operations centre (SOC) operations. Combined with data from 3D multi-person pose estimation and localisation algorithms running on the DARLENE machine learning Cloud, a dynamic 3D visual environment will be reconstructed, showing the location, orientation, affiliation and activity of occupants within the scene of the crime. In combination with accelerated 3D graphics powering a personalised identification friend or foe (IFF) signposting method, position and orientation data of the ARSGs will be used to render elements of the “live” 3D scene through a virtual point-of-view camera, allowing tactical squads to view their adversaries’ movements and orientations in real-time, even though solid walls, in a hyper-virtual representation of their surrounding environment ^31^. These visual cues, in combination with the verbal communication from the SOC, will allow tactical units to better formulate their neutralisation strategy, reduce or completely eliminate friendly fire casualties, and even utilise available ammunition more efficiently.

This pilot will take place at the Markopoulo Training Facility located in Athens, currently used for training purposes by LEAs and emergency responders since it provides a controlled and safe environment for both testing and validation. The facility’s block of buildings will be used and re-purposed, along with existing infrastructure (e.g. pre-installed CCTV cameras, telephone network and ethernet cables) for simulation purposes. The aim of this pilot will be to demonstrate the use of the DARLENE tools for tactical-based decision-making to enhance LEA ongoing SA and level of preparedness, so as to be cognizant of the surrounding environment and to stay vigilant to potential dangers. As such, DARLENE will be evaluated in terms of both direct methods (e.g. situational awareness global assessment technique ^32^ and questionnaires), as well as indirect methods (including observer rating instruments and performance outcome measures) pertaining to the tools’ efficacy to ensure and increase LEA survival rate and tactical decision-making when faced with potentially life-threatening situations.

## Targeted Technological Breakthroughs

### AR and wearable technologies for increased SA and improved individual functioning

Augmented reality (AR) presents an opportunity to respond to threats in an agile manner, breaking the “response to action” cycle of first suffering and reviewing latest attacks, and then mitigating and facilitating a response. Through the merits of AR, police officers could be capacitated to more rapidly process the OODA loop to react to unfolding events, thus gaining the upper hand against adversaries when dealing with incidents taking place in physical locations. With the use of AR smart glasses, law enforcement agents (LEAs) will deprive criminals and terrorists of the initiative, while simultaneously developing a common operating picture (COP) for both on-scene personnel and their command staff, one that is based on an aggregation of data collected from various sources. For this to work, DARLENE employs ML towards analysing large volumes of data acquired by multiple sensors in real-time, enabling assessments to be provided faster and in a much more coordinated manner in aid of LEA decision-makers, in order to enable them to outmanoeuvre the adversary. To accomplish this, DARLENE will develop ML methods for SA that are optimised for operating on wearable devices in real time while offering an improved user experience adapting visualisation to the officer’s psychophysical state and situation context. Eventually the goal is to use AR and wearable technologies in order to scale beyond the limits of the human capacity for processing and analysing scene information, and potentially even shining a light in areas undetectable by humans (blind spots).

In order to quantifiably measure the performance of the envisioned system, specific indicators have been specified toward obtaining reliable LEA performance outcome measures. These have been elaborated in the previous Section, pertaining to the specific evaluation criteria defined for each of the foreseen use cases, e.g.: (i) DARLENE being evaluated in terms of time reduction to detect and resolve incidents, thus enhancing the integrity of aviation and critical national infrastructure security; and (ii) performance outcome measures (specified in training) pertaining to the extent in which the proposed tools increase filed operative survival chances and tactical decision-making when faced with potentially life-threatening situations.

### Real-time, federated artificial intelligence network

As explained in the previous Section, DARLENE follows a multi-layered architecture with several artificial intelligence (AI) components in each layer. Starting from the Wearable Edge Computing Node (WECN) that the officer carries with him/her several computer vision and monitoring elements are implemented. Computer vision algorithms cover basic scene understanding modules for instance segmentation, namely identification of the types of objects and their contour in the scene as well as 2D pose estimation of humans, that is detection of body key-points and estimation of body configuration. Using this information for processing at a higher, semantic level, DARLENE supports activity recognition as well. Since real-time operation is critical in order to timely notify an officer, the focus on performance requires lightweight implementations of the respective AI models. In this respect, it is crucial to address the trade-off between semantic/instance segmentation performance and speed. Preliminary targets for the project foresee the WECN enabling above 20 frames-per-second image segmentation speed, while suffering marginal performance penalties (e.g., maximum 15% error margin in mean Intersection over Union, max. 10% error in detection accuracy, etc.) in order to make them suitable for the real-time LEA application purposes.

WECN is also responsible for the real-time adaptation of the content presented to the officer through the Heads-Up Display using machine learning to infer stress level and take into account the context of each situation. For instance, in a shooting event where officer’s safety is jeopardised and higher stress levels are likely to be detected, a minimal set of critical information will be visualised related to the position of friends and foes, to help the officer obtain a tactical advantage and not clutter his field of view with unnecessary information. On the contrary, more details will be visualised in a routine patrol operation where the officer has a calmer mental state and can comprehend and gain from more information. Similar AI and computer vision functionalities are supported on the Patrol Car Edge Node (PCEN) and the cloud counterpart of DARLENE, with computational limitations being gradually alleviated and more complex and accurate AI pipelines being available (see below). Essentially DARLENE will implement a federated, pyramidal AI network topology with AI analysis requests distributed among computational resources in order to get the most accurate results while maintaining real-time performance.

### Hybrid fog and cloud computing approach for real-time orchestration

While DARLENE’s multi-layered design can cope with computational complexity by off-loading processing to fog and cloud resources, this process can affect real-time performance due to communication delay and latency. To tackle this, DARLENE foresees the utilisation of 5G communication, thus securing adequate communication bandwidth for the exchange of rich multimedia content and simultaneously minimising any time overhead for data transmission. More specifically, 5G deployments at the pilot sites are expected to demonstrate the benefit of 5G for the specified scenarios regarding high-bandwidth (maximum aggregated total system bandwidth of minimum 0.7 Gb/s is targeted) and low latency (below 5ms) requirements, ensuring reliable and dependable Internet with “zero perceived” downtime for the envisioned services provision. Exploiting the 5G communication infrastructure, a dedicated orchestration module, as depicted in Figure 2, will be responsible for distributing processing requests along the edge, fog and cloud continuum, thus realising a hybrid computation architecture. The orchestrator’s goal will be to maintain real-time performance taking into account resources utilization. In this regard, aside typical utilization metrics for each computation node, the orchestrator will take scene context into account as provided by the artificial intelligence modules for activity recognition. This way the orchestrator will be able to put higher priority in computation requests coming from police officers facing emergencies.

## Ethical aspects

Aside the targeted technological breakthrough, DARLENE aims through concrete design choices to comply and promote ethical principles and relevant legislation. First of all, data storage requirements are minimized since processing is performed in real-time and there is no use of historical data or interconnection with police databases foreseen. In fact, this real-time operation in addition to the edge processing capabilities of DARLENE, confine the lifecycle of data both in space, i.e. on the edge side, and in time, thus minimizing privacy and data mismanagement risks. In the cases where data are transferred to other computational nodes on the edge or the cloud, this is mandated by law enforcement agency operational needs and performed using secure communications channels with encryption. There is also research work conducted in mitigating machine learning (ML) dataset bias particularly for activity recognition through the use of online learning and data augmentation techniques. Another ethical safeguard of DARLENE, is the exclusion of any obtrusive sensors such as infrared sensors or audio capturing devices from its ecosystem. Instead DARLENE relies primarily on sensor data from CCTV equipment, typically cameras, which have an established legal framework for their operation both under regular conditions, i.e. General Data Protection Regulation (GDPR) ^5^, and during a police operation, i.e. Police Directive ^6^. Transmission and processing of sensitive data as foreseen in DARLENE use cases, such as pictures of suspects sent by HQ or biometric data for personalized augmented reality content adaptation, are indeed regulated and allowed by the Police Directive. Finally, it should be also noted that any data collection for ML training or validation of the developed technology is monitored and supervised by an independent Ethics Committee, following a specific protocol for recruiting and informing human participants in experiments while the project is ethically scrutinized by the EC.

## Methods

### Wearable Edge Computing Node hardware capabilities

A crucial task within DARLENE is to define and gather together the DARLENE hardware and Internet of Things infrastructure (e.g., appropriate bio signals acquisition sensors, smart glasses, cameras etc.) so as to accommodate the processing capabilities that should be delivered by the Wearable Edge Computing Node (WECN). Central to the WECN’s design are the binocular augmented reality smart glasses (ARSGs), which will be based on a custom version of the “Talens Holo” smart glasses, manufactured (e.g., optics, electronics, software, and frame development) specifically for the DARLENE consortium by project partner Youbiquo srl. To guide the design of the hardware components, the existing “Talens Holo” platform has been used as a benchmark to identify further platform-specific considerations and inform the specifications of the DARLENE smart glasses prototype. In order to examine efficient means to combine local and remote computations for the purposes of real-time performance on the smart glasses, additional computation devices (e.g., NVIDIA Jetson micro-processing module) are integrated as part of the WECN solution specification, utilised to offload computations in close proximity to the user. As a result, the WECN is envisioned as a modular solution comprised of: (i) a head module (ARSGs, complete with helmet) based on a high-performant mobile platform with integrated graphics processor to emulate capacities of high-end Android smartphones; (ii) a Belt module equipped with an NVDIA Jetson microprocessor for artificial intelligence applications; and (iii) a strap module equipped with Electrocardiography and Accelerometer sensors. Additional deployment considerations (e.g., placement of the machine learning modules, capacity to run the Heads-Up Display applications as a standalone .apk package on the smart glasses, etc.) will be explored as the project progresses over its lifetime until 2023

### Instance segmentation for detecting humans and objects of interest

**Figure 4.**
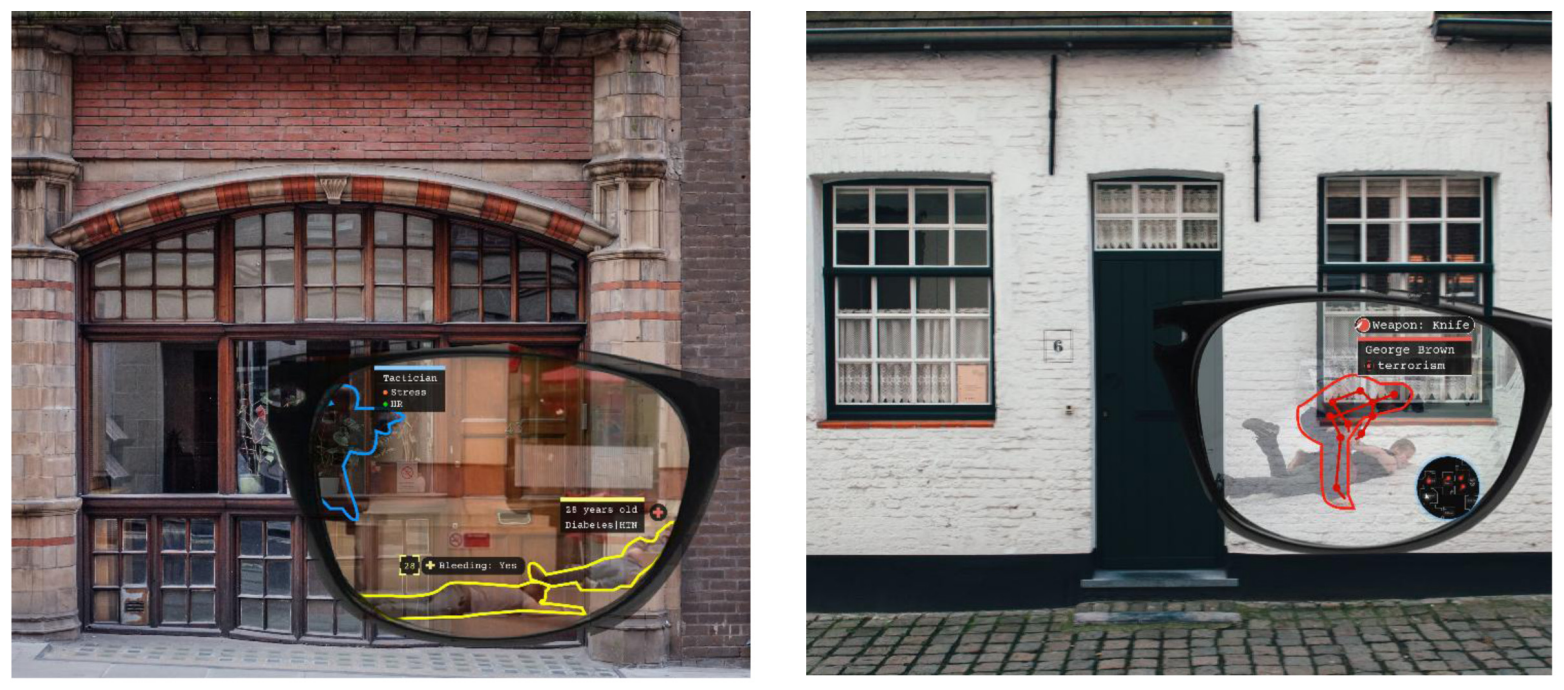
Use Case 2 mock-up screenshots.

A core functionality for DARLENE, as is also highlighted in Figure 3 and Figure 4, is the detection of humans and objects in video data coming either from the augmented reality (AR) smart glasses (ARSGs) or other Internet of Things cameras in the DARLENE ecosystem. To this end, video streams are decoded to extract consecutive frames that are then processed with an instance segmentation method. Instance segmentation is a computer vision task where objects in the scene are segmented from the background and are labelled according to the class they belong to. In DARLENE, these classes include humans and objects like firearms, vehicles and unattended luggage that are of particular interest for law enforcement officers. To verify the performance levels of state of the art, initial tests were carried out during October 2020 on a subset of 34 videos from the RWF-2000 dataset for violence detection ^33^. This dataset was selected since it contains video footage from surveillance cameras capturing violent acts that are clearly of relevance to both of the foreseen use cases, as these have already been described. Concretely, we have tested the Mask R-CNN method for instance segmentation ^34^, one of the top performing methods based on a convolutional network pipeline. Mask R-CNN uses a convolutional network to extract features from an image and subsequently a region proposal network is applied to detect bounding boxes that potentially contain an object of interest. Afterwards, bounding boxes are filtered and the final branch of Mask R-CNN produces the mask (i.e. boundary) of an object inside a bounding box. The final outputs of Mask R-CNN are the bounding box, object category and mask for each object in an image.

### 2D pose estimation of humans

Another basic functionality for DARLENE is 2D pose estimation which refers to the computation of the spatial locations of body joints for each human in an image. It will help officers to better analyse ongoing events even behind concrete walls as shown in the mock-up of Figure 4 and will also enable higher level computer vision tasks for human activity recognition and tracking, thus having a central role in the second use case of DARLENE “Tactical neutralization of human adversaries in the presence of friendlies” as it will help in distinguishing friends from foes and understanding their intention.

We have tested the HigherHRNet method ^18^ that has shown impressive results on the MS-COCO dataset ^35^, using the same 34 videos as in the previous section. Since evaluation metrics cannot be produced due to lack of relevant ground truth data in the RWF-2000 dataset, only qualitative results are depicted. HigherHRNet follows a multi-resolution convolutional architecture where separate branches process representations of an image at different scales, whereas intermediate processing outputs are fused using upsampling and downsampling. The output of HigherHRNet is a set of heatmaps for each person on an image, where each heatmap provides the location of a body joint (e.g. left shoulder, right arm etc.).

### DARLENE concept validation with end-users

In order to proceed with a first validation of the DARLENE concept and use cases, three online co-creation workshops ^36^ with end users were organised in November 2020, carried out via teleconferencing software. Participants were invited to the workshop, via emails to the contacts of DARLENE law enforcement agency partners. Prior to their participation, all participants were informed in writing by the workshop organizers about the research activity, the purpose of the study and of data collections, the collected personal data and their rights, Furthermore, all participants signed the informed consent document, prior to the workshops.

A small number of end-user participants was scheduled for each workshop day, in order to achieve an efficient and effective online collaboration, reaching a total of 30 participants in all workshops. The workshops involved structured co-creation activities for eliciting user needs and preferences. The workshops were facilitated by a user experience researcher, with considerable experience in participatory design activities.

All workshops had the same structure of activities. In particular, each workshop was structured in five (5) main sections:

1. Discussion of the aims and objectives of the workshop, and provision of general instructions to participants.
2. Warm-up activity, acting as an ice-breaker to stimulate discussions within the group.
3. Presentation of DARLENE and its use cases, to familiarize participants with the project.
4. Co-creation activities for each use case, aiming to identify functionality that participants would like the DARLENE technologies to have.
5. Workshop evaluation.

Activities were delivered online, and participants’ input was annotated by the facilitator on a shared electronic board, available to all participants. No video or audio recordings were made. The workshop outcomes were analysed manually, following a combination of deductive and inductive coding, involving two researchers ^37^. In particular, one code for each one of the functionalities identified in the desired functionality activity was created, following the deductive coding approach. Then, the researchers examined the data regarding functional requirements in order to assign one of the predefined codes. In the cases when the need for assigning a new code was identified, this was added to the set of codes, and all responses were re-examined, following the inductive coding approach. The examination of responses and code assignment was carried out by two individual researchers. The outcomes of the two individual analyses were compared, following a consensus-building approach to address inconsistencies the codes assigned.

### Enhancing LEAs situational awareness through augmented reality

In order to leverage the situational awareness of law enforcement agents (LEAs) in the field an agile decision-making mechanism regarding the visual cues that will be provided to the LEAs through the augmented reality (AR) glasses, has been developed. This mechanism is responsible for the AR annotations displayed in front of LEAs’ field of view, and must keep the balance between (a) on one hand what should be displayed in order to help them be aware about what is happening around and (b) on the other hand prevent information overloading that might constitute a burden to their timely and efficient decisions and actions, which is a prominent requirement for tackling unlawful activities such as terrorist attacks. The decision-making mechanism takes into account several parameters that constitute LEAs context of use, such as their experience regarding their job, their stress level, as well as environmental conditions as for example the crowdedness of the environment. The mechanism combines an ontology modelling approach and combinatorial optimisation, towards reasoning about what information to present, when to present it, where in the AR display and how, taking into consideration the current context as well as display placement constraints. The objective is to optimise the situational awareness associated with the displayed AR user interface (UI), while at the same time avoiding information overload and induced stress.

Currently, the decision-making mechanism has been assessed following a formative expert review process, involving 10 experts, and in particular 5 user experience experts and 5 LEA experts. The evaluation process was split in two phases. During the first phase the law enforcement experts practitioners were requested to rank a list of predefined graphical user interface (GUI) components that would be used to provide information on the Heads-Up Displays (HUDs), according to their importance with regard to two tactical operation tasks (patrolling and incident resolution). Each expert returned an ordered list of GUI components, with components ranked in order of importance. A final ranking score for each component was calculated as the average of the individual scores. The resulting final ranking was used by the decision-making mechanism for inferring judgements regarding the placement and the Level of Detail of the components.

The second phase involved all 10 experts, who were provided with a number of diverse potential scenarios, based on the use cases which are detailed in the "Use Cases" section, illustrating how the detected scene elements (e.g., perpetrators, suspicious objects, etc.) are being outlined and relevant information (e.g., identified dangerous object details, top priority alerts, etc.) is being annotated on the HUDs. For each scenario assessed, experts were informed about the parameters according to which the decision-making mechanism provided the final visual information, such as the stress level of the officer, the environmental conditions, the officer's experience, etc.

## Results

### Instance segmentation for detecting humans and objects of interest

**Figure 5.**
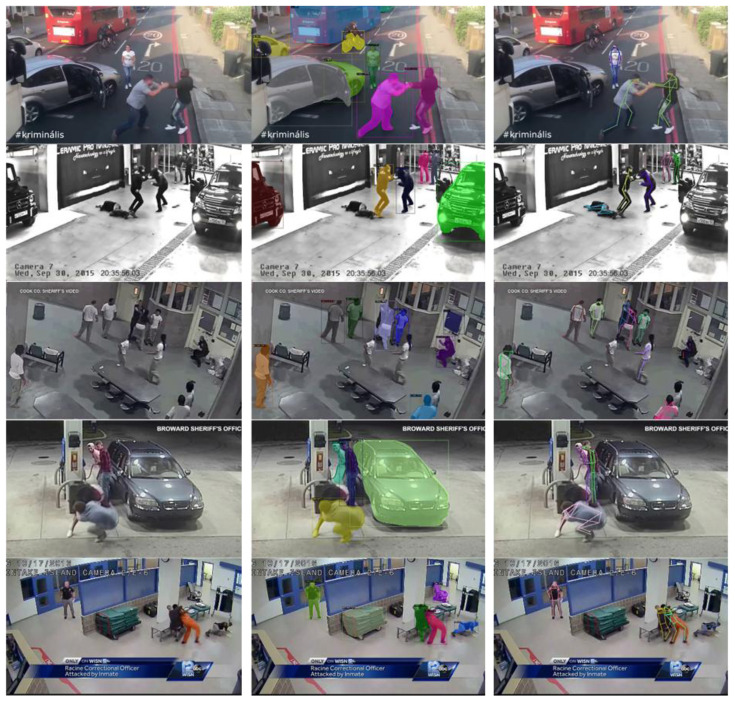
Qualitative results on indicative samples for the Violent Act dataset.

Qualitative results are depicted in Figure 5 where the left column of the figure shows the original frame and the middle column contains instance segmentation results, namely the detected objects’ bounding boxes and masks. Based on these examples, the quality of the results is good enough to support the foreseen functionalities of DARLENE for improved situational awareness and enhanced decision making, particularly as required by the first use “Rapid visual scene analysis for anomaly detection” since instance segmentation by detecting and separating object of interest from the background allow the rendering of relevant information on the officer field of view. Nonetheless there is still room for improvement as there are missed objects and false positives particularly in cases of heavily cluttered scenes that often occur during emergencies and security incidents. Such failure cases are depicted in the 2 ^nd^ and 3 ^rd^ row of the middle column of Figure 5 where some of the individuals in the scene are not detected presumably because of their unusual body configuration in the 2 ^nd^ row and partial occlusion by the table in the 3 ^rd^ row. There are also other objects that were not detected like the partially visible vehicle in 2 ^nd^ row and the table in the 3 ^rd^ one.

### 2D pose estimation of humans

The results of Figure 5 depict the body configuration for each person after the computed heatmaps are merged in a skeleton representation. The results are promising but there is substantial room for improvement, particularly for body configurations that are infrequent (e.g. humans lying on the ground) or are partially visible due to (self)-occlusions (e.g. cluttered scenes with multiple humans as in 3 ^rd^ row of Figure 5). Aside scene complexity, computational complexity is another critical factor, since DARLENE requires near real-time performance for the timely visualisation of analysis results on the augmented reality glasses. To accomplish this given the complexity of the foreseen computational tasks and the limited resources of the Wearable Edge Computing Node, there will be inevitably a trade-off between quality of results and computation time as none of the tested methods operates in real time. To this end new deep architectures will be explored that can optimally utilise available resources and produce in real time results without degrading much of the quality.

### DARLENE concept validation with end-users

Overall, participants expressed positive feedback for the overall concept and rich comments, ideas, and feedback for the use cases involved, attesting their interest in the foreseen technologies and identifying the lack of existing approaches in current practices. The systematic analysis of the outcomes of the workshops constituted the foundation for specifying the system requirements, resulting in 44 requirements. Furthermore, it identified the Graphical User Interface (GUI) components that should be implemented for addressing the reported needs. As explained in the following section, the GUI components were then evaluated in realistic scenarios by the project's law enforcement practitioners.

### Formative evaluation of the SA decision-making mechanism

The results of this evaluation were very encouraging regarding the efficiency of the devised mechanism. All experts agreed that the components that the decision-making mechanism decided to visualize were necessary and appropriate in all the use cases that were explored. In certain cases, suggestions for the information that should be depicted were made (e.g. which icons would be appropriate to use in a particular case). Currently, in the context of the DARLENE project, larger scale evaluations are being planned to be organised with law enforcement agents, so that the decision-making mechanism is assessed in realistic conditions, such as the airport of Larnaca Cyprus.

## Discussion

As DARLENE is at its initial phase, much of the presented technology is still under development and substantial integration efforts are required to produce an end-to-end framework. Nonetheless, a clear roadmap for the realization of the DARLENE ecosystem has been defined, whereas preliminary results are quite promising. In this effort, a major research milestone is the successful integration of a Wearable Edge Computing Node (WECN) with the augmented reality (AR) smart glasses (ARSGs) and other wearable sensors that will constitute the initial prototype for the *in situ* deployment of DARLENE. A prerequisite to this is to meet (near-) real time performance requirements for computer vision (CV) and machine learning (ML) modules on the WECN while achieving a high accuracy rate in order to foster the adaptive AR capabilities of the ARSGs. Another important goal is the setup of the 5G infrastructure and the integration with the fog and cloud side of the ecosystem enhanced with all the foreseen artificial intelligence functionalities. Individuals research goals include rapid scene analysis via CV modules and communication delay minimisation via the creation of a feedback loop between the 5G orchestrator and ML modules output. Another key technology for DARLENE is the digitisation, i.e. 3D reconstruction, of large buildings or other kind of potential operation theatres for law enforcement agents (LEAs) which is the first step for the creation of an advanced command and control framework that will provide a complete and live image of an operation to agency headquarters.

Aside technological achievements, an important factor for the success of DARLENE is the acceptance of the envisioned technologies by end users. In this respect, findings from preliminary assessments with target users are encouraging, exhibiting the positive attitude of LEAs, a result which is aligned with recent research ^38^. Future work will aim to address limitations of the current study that has been conducted for assessing the SA decision-making mechanism, involving a limited number of user experience and LEA experts, by conducting field studies with representative end users. Despite its limitation, however, this study has proved highly valuable in identifying issues that should be addressed prior to evaluating the DARLENE technology with end users.

Finally, the success of DARLENE is highly dependent on establishing a robust ethical framework in line with existing legislation and ethics principles both during its development and deployment. To this end, DARLENE development is continuously scrutinised by internal and external ethics’ experts aiming at substantial progress over current practices via *in situ*, i.e. edge, processing and real-time performance that will minimise data storage requirements and ethical concerns. DARLENE also foresees specific steps in order to create a community of LEA end-users, facilitate any uptake of the technology and ensure its alignment with user requirements.

## Conclusions

In this paper we presented the DARLENE ecosystem for next generation smart policing through the combination of augmented reality (AR) and artificial intelligence (AI). DARLENE aims to enhance officers’ capabilities on the field allowing the timely analysis of multiple data streams via a scalable AI architecture and the personalised, adaptive presentation of AI results through AR. In this paper, we have provided details on the current technological landscape that DARLENE builds on and the core technologies that will be further advanced. A first look on the DARLENE architecture has been also given, with details on the foreseen use cases. We have further elaborated on the scientific advances that must be achieved so that DARLENE realises its goal and provided insight on the research directions that will be pursued within the project.

## Data availability

The work reported in this paper has been partially based on desk research in publicly available resources. These resources have been cited within the article. Data that has been collected through co-creation workshops and interviews with experts cannot be made publicly available, due to privacy and ethical restrictions. In addition, by design, the study did not conduct audio or video recordings, and as such transcriptions of participants’ quotes cannot be provided.

Please contact George Margetis ( gmarget@ics.forth.gr) in order to request access to the data, explaining the research in the context of which the data is requested, how data will be used, and who will have access to it. All requests should be accompanied by a full CV of the person requesting the data, and the CVs of anyone who will be given access to the data., as well as by a confirmation that the person has no conflict of interest and will not share the provided data with any third parties.

## Software availability

The source code of the DARLENE project cannot be made openly available since it regards sensitive information for law enforcement agencies. As such, many of the deliverables regarding the project’s results are classified as confidential or EU restricted.

Please contact George Margetis ( gmarget@ics.forth.gr) in order to request access to the data, explaining the research in the context of which the data is requested, how data will be used, and who will have access to it. All requests should be accompanied by a full CV of the person requesting the data, and the CVs of anyone who will be given access to the data., as well as by a confirmation that the person has no conflict of interest and will not share the provided data with any third parties.

## Footnotes

^1^ https://www2.deloitte.com/content/dam/Deloitte/uk/Documents/public-sector/deloitte-uk-future-of-policing.pdf

^2^ https://www.abiresearch.com/press/smart-glasses-set-for-significant-enterprise-not-c/

^3^ Directive 95/46/EC of the European Parliament and of the Council of 24 October 1995 on the protection of individuals with regard to the processing of personal data and on the free movement of such data.

^4^ Regulation (EU) 2016/679 of the European Parliament and of the Council of 27 April 2016 on the protection of natural persons with regard to the processing of personal data and on the free movement of such data, and repealing Directive 95/46/EC (General Data Protection Regulation)

^5^ Regulation (EU) 2016/679 of the European Parliament and of the Council of 27 April 2016 on the protection of natural persons with regard to the processing of personal data and on the free movement of such data, and repealing Directive 95/46/EC (General Data Protection Regulation), European Law Identifier (ELI): http://data.europa.eu/eli/reg/2016/679/2016-05-04

^6^ Directive (EU) 2016/680 of the European Parliament and of the Council of 27 April 2016 on the protection of natural persons with regard to the processing of personal data by competent authorities for the purposes of the prevention, investigation, detection or prosecution of criminal offences or the execution of criminal penalties, and on the free movement of such data, and repealing Council Framework Decision 2008/977/JHA, European Law Identifier (ELI): http://data.europa.eu/eli/dir/2016/680/2016-05-04
