# Supplementary material for: DARLENE – Improving situational awareness of European law enforcement agents through a combination of augmented reality and artificial intelligence solutions
Source: Open Res Eur. 2022 Jan 21;1:87. Originally published 2021 Jul 30. [Version 2] doi: 10.12688/openreseurope.13715.2 (PMC10446065; doi:10.12688/openreseurope.13715.2)
Supplement: Peer review history

# Peer review: Reviewer response for version 2

**DOI:** 10.21956/openreseurope.15584.r28421

**Published:** 2022-01-28

**Authors:** Anastasios Delopoulos

**Affiliations:** 1 Multimedia Understanding Group, Information Processing Laboratory, Department of Electrical and Computer Engineering, Aristotle University of Thessaloniki, Thessaloniki, Greece

After reading the revised version of the manuscript I think that the authors have responded to my comments in a satisfactory way.

Is the study design appropriate and does the work have academic merit?

Yes

Is the work clearly and accurately presented and does it cite the current literature?

Yes

If applicable, is the statistical analysis and its interpretation appropriate?

Not applicable

Are all the source data underlying the results available to ensure full reproducibility?

Yes

Are the conclusions drawn adequately supported by the results?

Yes

Are sufficient details of methods and analysis provided to allow replication by others?

Partly

Reviewer Expertise:

NA

I confirm that I have read this submission and believe that I have an appropriate level of expertise to confirm that it is of an acceptable scientific standard.

# Peer review: Reviewer response for version 2

**DOI:** 10.21956/openreseurope.15584.r28422

**Published:** 2022-01-24

**Authors:** Stylianos Asteriadis

**Affiliations:** 1 Department of Data Science and Knowledge Engineering, Maastricht University, Maastricht, The Netherlands

I am happy with the revisions the authors applied on the paper. Any additional, quantified piece of information, especially regarding user acceptance and requirements, would be more than welcome. Otherwise, I think the paper is very thorough in communicating the overall objectives, goals and subgoals of the project, also with regards to multiple aspects of the state of the art in ICT.

Is the study design appropriate and does the work have academic merit?

Yes

Is the work clearly and accurately presented and does it cite the current literature?

Yes

If applicable, is the statistical analysis and its interpretation appropriate?

Not applicable

Are all the source data underlying the results available to ensure full reproducibility?

No source data required

Are the conclusions drawn adequately supported by the results?

Yes

Are sufficient details of methods and analysis provided to allow replication by others?

Partly

Reviewer Expertise:

Computer Vision, Artificial Intelligence, Automated Human Behaviour Recognition, Affective Computing

I confirm that I have read this submission and believe that I have an appropriate level of expertise to confirm that it is of an acceptable scientific standard.

# Peer review: Reviewer response for version 1

**DOI:** 10.21956/openreseurope.14789.r27918

**Published:** 2021-11-17

**Authors:** Anastasios Delopoulos

**Affiliations:** 1 Multimedia Understanding Group, Information Processing Laboratory, Department of Electrical and Computer Engineering, Aristotle University of Thessaloniki, Thessaloniki, Greece

The manuscript introduces the concept of DARLENE, a novel framework for supporting law enforcement agencies (LEAs), particularly in the field by means of Artificial Intelligence (AI) and Augmented Reality (AR). The key functionality of the proposed system relies on the analysis of visual content and signals captured by wearable sensors in order (i) to support real time decision making from officers acting in the field as well as (ii) to provide access to timely information regarding the crime scene and the involved persons. The manuscript offers a very good review of state of the art technologies that have been so far used or proposed for similar systems. The proposed solution intends to outperform SoA in terms of privacy protection, accuracy in decision making and timelines. Although the success towards these goals cannot be judged at this early concept building stage, the manuscript reports the positive results regarding acceptance of the system from end users.

 This reviewer has the opinion that the text needs to clarify/provide additional info on the following issues:

1. The authors claim that DARLENE will offer a solution that will comply to GDPR restrictions. As it is very accurately stated "especially concerning the use of ARSGs by LEAs, and the integration of sensing hardware, such as cameras and microphones, AR gear is seen as having the capacity to record video and audio in a discreet manner, raising the issue that people being recorded may actually not even be aware of it. The image of a person constitutes personal data within the meaning of Article 2(a) of Directive 95/46/EC3 and of Article 4(1) of the GGDPR4, inasmuch as it allows for the identification of individuals"; the authors have to explain the exact way this critical issue will be solved by the edge processing methodology that DARLENE is expected to offer.
2. Much of the success (accuracy, speed) of the proposed framework depends on the edge processing solutions that essentially rely on the capabilities of the Wearable Edge Computing Node (WECN). Since this is a critical component a more detailed description on its architecture and its processing core must be included in the text. What are the hardware options considered by the research team?
3. Although the text offers a very interesting qualitative presentation of the various components, it is highly recommended to include quantification of critical parameters that may influence the design: (a) Processing needs for the edge devices and for the cloud services (per user stream), (b) Necessary network bandwidth and latency limits, (c) Acceptable accuracy of the involved detection modules etc.

Is the study design appropriate and does the work have academic merit?

Yes

Is the work clearly and accurately presented and does it cite the current literature?

Yes

If applicable, is the statistical analysis and its interpretation appropriate?

Not applicable

Are all the source data underlying the results available to ensure full reproducibility?

Yes

Are the conclusions drawn adequately supported by the results?

Yes

Are sufficient details of methods and analysis provided to allow replication by others?

Partly

Reviewer Expertise:

Signal and Image Processing, Computer Vision, Machine Learning, Behavioral Informatics

I confirm that I have read this submission and believe that I have an appropriate level of expertise to confirm that it is of an acceptable scientific standard, however I have significant reservations, as outlined above.

# Response

**Published:** 2022-01-11

**Authors:** George Margetis

**Affiliations:** 180580, Greece

***Reviewer's comment: **The manuscript introduces the concept of DARLENE, a novel framework for supporting law enforcement agencies (LEAs), particularly in the field by means of Artificial Intelligence (AI) and Augmented Reality (AR). The key functionality of the proposed system relies on the analysis of visual content and signals captured by wearable sensors in order (i) to support real time decision making from officers acting in the field as well as (ii) to provide access to timely information regarding the crime scene and the involved persons. The manuscript offers a very good review of state of the art technologies that have been so far used or proposed for similar systems. The proposed solution intends to outperform SoA in terms of privacy protection, accuracy in decision making and timelines. Although the success towards these goals cannot be judged at this early concept building stage, the manuscript reports the positive results regarding acceptance of the system from end users.*

**Answer: **We would like to thank the reviewer for the constructive comments and encouraging remarks.

***Reviewer's comment: **This reviewer has the opinion that the text needs to clarify/provide additional info on the following issues:*

- *The authors claim that DARLENE will offer a solution that will comply to GDPR restrictions. As it is very accurately stated "especially concerning the use of ARSGs by LEAs, and the integration of sensing hardware, such as cameras and microphones, AR gear is seen as having the capacity to record video and audio in a discreet manner, raising the issue that people being recorded may actually not even be aware of it. The image of a person constitutes personal data within the meaning of Article 2(a) of Directive 95/46/EC3 and of Article 4(1) of the GGDPR4, inasmuch as it allows for the identification of individuals"; the authors have to explain the exact way this critical issue will be solved by the edge processing methodology that DARLENE is expected to offer.*

**Answer: **An additional section titled ‘Ethical aspects’ has been added in which ethical concerns are addressed in more detail.

***Reviewer's comment: ***

- *Much of the success (accuracy, speed) of the proposed framework depends on the edge processing solutions that essentially rely on the capabilities of the Wearable Edge Computing Node (WECN). Since this is a critical component a more detailed description on its architecture and its processing core must be included in the text. What are the hardware options considered by the research team?*

**Answer: **We have added an additional paragraph in the ‘Methods’ section, in which we describe the WECN in more detail (incl. the hardware considered for its implementation).

***Reviewer's comment: ***

- *Although the text offers a very interesting qualitative presentation of the various components, it is highly recommended to include quantification of critical parameters that may influence the design: (a) Processing needs for the edge devices and for the cloud services (per user stream), (b) Necessary network bandwidth and latency limits, (c) Acceptable accuracy of the involved detection modules etc.*

**Answer: **We have added some performance evaluation metrics and target values which we consider as success criteria for the outputs of the project. These have been integrated in the ‘Targeted Technological Breakthroughs’ section, for each of the technologies mentioned therein.

# Peer review: Reviewer response for version 1

**DOI:** 10.21956/openreseurope.14789.r27917

**Published:** 2021-11-08

**Authors:** Stylianos Asteriadis

**Affiliations:** 1 Department of Data Science and Knowledge Engineering, Maastricht University, Maastricht, The Netherlands

This article provides a high level description of the DARLENE EU project, which is now at its initial stages. The project is a very ambitious action, aspiring to make use of the latest advances in AI and AR for facilitating the job of LEAs (Law Enforcement Agencies) in critical  circumstances requiring intervention of police forces. The paper is very well written, covering the state of the art and discussing every aspect of the technologies used. The authors are covering the latest literature and all the components involved are very clearly explained. Besides  readability, once DARLENE becomes a working system, its impact on the actual work of LEAs can be very significant, while it can become a starting point for AR/AI technologies becoming widely available  in crime, safety and precaution.

 Given the nature of the article, as well as the fact that the project is at its initial stages, it is not possible to judge on an easy replication of the methods described. The authors may want to  update the article by focusing on the following two aspects: 1. More detailed outcomes of the requirement extraction steps and, 2. They could provide more  details on the methods/public data they already used and link these methods to the actual use cases of the project. 

 Apart from the abovementioned comments, some less minor pieces of advice could be the following:

 Abstract: A smoother introduction regarding what DARLENE is, and what kind of applications it addresses, maybe through examples, would be good to have. This would make clear from the beginning what the role of AI, AR, and Computer Vision, is (e.g. activity recognition, posture analysis, etc.) in the project.

 Introduction: In the first paragraph, The passing from AR to ML can be made smoother. An introductory paragraph summarizing the usefulness of each of the employed technologies within the context of the proposed work/use cases and in relation with each other would add to the readability of the work. This information is indeed provided later in the article, but an earlier explanation of how these concepts come together concretely, would be beneficial, in my view.

 Can the authors provide a discussion on the ethical aspects of using AI beyond just data privacy? For example, what happens with biases, false positives or negatives, could be further discussed.

 Another minor comment is that the authors are making probably too frequent use of acronyms. It is understood that in a highly sophisticated system like the one presented in the article, this may be useful. I would suggest, though, that acronyms are re-written in full the first time they are used in each section. An index of acronyms could also help.

Is the study design appropriate and does the work have academic merit?

Yes

Is the work clearly and accurately presented and does it cite the current literature?

Yes

If applicable, is the statistical analysis and its interpretation appropriate?

Not applicable

Are all the source data underlying the results available to ensure full reproducibility?

No source data required

Are the conclusions drawn adequately supported by the results?

Yes

Are sufficient details of methods and analysis provided to allow replication by others?

Partly

Reviewer Expertise:

Computer Vision, Artificial Intelligence, Automated Human Behaviour Recognition, Affective Computing

I confirm that I have read this submission and believe that I have an appropriate level of expertise to confirm that it is of an acceptable scientific standard, however I have significant reservations, as outlined above.

# Response

**Published:** 2022-01-11

**Authors:** George Margetis

**Affiliations:** 180580, Greece

***Reviewer's comment:** This article provides a high level description of the DARLENE EU project, which is now at its initial stages. The project is a very ambitious action, aspiring to make use of the latest advances in AI and AR for facilitating the job of LEAs (Law Enforcement Agencies) in critical  circumstances requiring intervention of police forces. The paper is very well written, covering the state of the art and discussing every aspect of the technologies used. The authors are covering the latest literature and all the components involved are very clearly explained. Besides  readability, once DARLENE becomes a working system, its impact on the actual work of LEAs can be very significant, while it can become a starting point for AR/AI technologies becoming widely available  in crime, safety and precaution.*

**Answer: **We would like to thank the reviewer for the thorough review, encouraging comments and insightful remarks, that help our paper better reflect the work carried out.

***Reviewer's comment: **Given the nature of the article, as well as the fact that the project is at its initial stages, it is not possible to judge on an easy replication of the methods described. The authors may want to  update the article by focusing on the following two aspects: 1. More detailed outcomes of the requirement extraction steps and, 2. They could provide more  details on the methods/public data they already used and link these methods to the actual use cases of the project.  Regarding (1), we have added a new paragraph in the ‘DARLENE ecosystem’ section, where we elaborate on the requirements extraction procedures carried out with LEA partners joining the DARLENE project. Regarding (2), additional details that more clearly demonstrate the links between the ML algorithms and project use cases have been added in the ‘Methods’ and ‘Results’ sections.*

*Apart from the abovementioned comments, some less minor pieces of advice could be the following:*

*Abstract: A smoother introduction regarding what DARLENE is, and what kind of applications it addresses, maybe through examples, would be good to have. This would make clear from the beginning what the role of AI, AR, and *Computer Vision, is (e.g. activity recognition, posture analysis, etc.) in the project.

**Answer: **The Abstract has been revised to reflect this particular concern of the reviewer.

***Reviewer's comment:** Introduction: In the first paragraph, The passing from AR to ML can be made smoother. An introductory paragraph summarizing the usefulness of each of the employed technologies within the context of the proposed work/use cases and in relation with each other would add to the readability of the work. This information is indeed provided later in the article, but an earlier explanation of how these concepts come together concretely, would be beneficial, in my view.*

**Answer: **We have added the following statement: “AR alone however cannot deal with the complex task of scene interpretation, as it is a technology primarily focused on how to optimally combine digital and virtual information in one seamless representation.” which we think provides a smoother “bridge” between the AR and ML introductory sections.

***Reviewer's comment:*** *Can the authors provide a discussion on the ethical aspects of using AI beyond just data privacy? For example, what happens with biases, false positives or negatives, could be further discussed.*

**Answer: **An additional section titled ‘Ethical aspects’ has been added in which ethical concerns are addressed.   

***Reviewer's comment:** Another minor comment is that the authors are making probably too frequent use of acronyms. It is understood that in a highly sophisticated system like the one presented in the article, this may be useful. I would suggest, though, that acronyms are re-written in full the first time they are used in each section. An index of acronyms could also help.*

**Answer: **We have significantly decreased the amount of abbreviations used throughout the text, and where appropriate, we have opted to define abbreviations at the first occurrence in each section, as the reviewer suggests.
